# A Statistical Model to Determine Biomechanical Limits for Physically Safe Interactions With Collaborative Robots

**DOI:** 10.3389/frobt.2021.667818

**Published:** 2022-02-03

**Authors:** R. Behrens, G. Pliske, M. Umbreit, S. Piatek, F. Walcher, N. Elkmann

**Affiliations:** ^1^ Robotic Systems, Fraunhofer IFF, Magdeburg, Germany; ^2^ Department of Trauma Surgery, Otto von Guericke University, Magdeburg, Germany; ^3^ BGHM, Mainz, Germany

**Keywords:** safety, physical human-robot interaction, biomechanical limits, collision, onset of pain, impact, pinching

## Abstract

Collaborative robots (cobots) provide a wide range of opportunities to improve the ergonomics and efficiency of manual work stations. ISO/TS 15066 defines power and force limiting (PFL) as one of four safeguarding modes for these robots. PFL specifies biomechanical limits for hazardous impacts and pinching contacts that a cobot must not exceed to protect humans from serious injuries. Most of the limits in ISO/TS 15066 are preliminary, since they are based on unverified data from a literature survey. This article presents a human-subject study that provides new and experimentally verified limits for biomechanically safe interactions between humans and cobots. The new limits are specifically tailored to impact and pinching transferred through blunt and semi-sharp surfaces as they can occur in the event of human error or technical failures. Altogether 112 subjects participated in the study and were subjected to tests with emulated impact and pinching loads at 28 different body locations. During the experiments, the contact force was gradually increased until the load evoked a slightly painful feeling on the subject’s body location under test. The results confirm that the pain thresholds of males and females are different in specific body regions. Therefore, when defining biomechanical limits, the gender difference must be taken into account. A regression model was utilized to incorporate the gender effect as a covariate into a conventional statistical distribution model that can be used to calculate individual limits, precisely fitted to a specific percentile of a mixed group of male and female workers which interacting with cobots.

## 1 Introduction

Collaborative robots (cobots) implement a physical form of human-robot interaction (pHRI) in industrial manufacturing to bridge the gap between full automation and manual labor (Krüger et al., 2009; Chen et al., 2014; Michalos et al., 2014; Antonelli et al., 2016). The general concept of pHRI in manufacturing is to assign assembly tasks to robots, which require endurance, speed, and accuracy, while all other tasks remain with the human, especially those that require dexterity, experience, or the ability to solve complex problems (Helms et al., 2002; Michalos et al., 2014; Michalos et al., 2015). Such a division of labor between humans and robots has great potential to improve working conditions (Busch et al., 2017; Pearce et al., 2018; Makrini et al., 2019). Another advantage is that costly and space consuming safety sensors or fences are no longer needed (Bloss, 2016). Despite the sophisticated safety features modern cobots have today, their installation in industrial facilities requires additional efforts to reduce the injury risks from impacts or pinching contacts (Alami et al., 2006; de Santis et al., 2008; Haddadin and Croft, 2016).

ISO/TS 15066 specifies the safety requirements for cobots operating in industrial environments. According to this standard, any unintended human-robot contact needs be considered as a hazard, which must not cause biomechanical stress beyond the onset of pain. Injuries, even slight ones, are not allowed at all. As a metric to evaluate the injury risk, ISO/TS 15066 provides a list of biomechanical limits for 29 different body locations. One part of the limits stems from a study with 100 human subjects (Melia et al., 2019). Given the design of the study, the pressure-based limits apply only to contact situations in which a semi-sharp piece of the robot clamps the human body. The second part sets limits for impacts and pinching contacts over blunt surfaces. Since they were estimated from literature data, they are considered preliminary and will be replaced once more reliable values become available (ISO/TS 15066, 2016).

The literature research at the beginning of our study revealed that especially limits for the prevention of pain or even slight injuries are rather rare. It turned out that other studies have either examined only a few regions of the human body and/or insignificant small subject groups (Patrick, 1981; Dhaliwal et al., 2002; Muggenthaler et al., 2006; Povse et al., 2011). Most of the studies on the biomechanical consequences of impacts utilized a pendulum as a testing system to apply impact loads to human subjects. Due to the small samples and limited scope, the data from these studies do not allow deriving reliable limits for the entire human body or even particular body regions. Unlike impacts, the consequences of pinching contacts have been examined in several studies using algometry (Yamada et al., 1996; Saito and Ikeda, 2005; Melia et al., 2014). As mentioned before, an extraordinary study in terms of sample size is the one of Melia et al. Altogether, the pain thresholds of 100 subjects have been measured at 29 body locations with pinching forces. Unlike other studies with a similar scope, Melia et al. focused on limits based on peak pressures, instead of maximum forces. Park et al. (2019) repeated their experiments with 90 male subjects, but only on 15 body locations.

The few results from the literature survey show that the available amount of data from experiments with human-subjects is insufficient to specify new limits, which can be used to replace the preliminary ones in ISO/TS 15066. Data from cadaver or animal studies are not a suitable alternative, since they are frequently drawn from experiments with intense loads that typically cause fatal injuries. This article reports on a human-subject study that delivers biomechanical limits, experimentally ascertained in load tests with 112 human subjects. Unlike most other studies, we specifically tailored the design of our experiments to the peculiarities of robots and the requirements of safe pHRI in industrial environments.

This article is structured as follows: Section 2 describes the methods and materials employed in the study, detailing the experiments, their design, the systems used and the procedures applied. Section 3 presents the results obtained in the experiments and analyzes them with statistical methods. Key of this section is the development of a distribution model that can be used to set limits for mixed-gender groups. The study’s methods and results are discussed in Section 4 and compared with the findings of related studies. Section 5 concludes our work and gives brief insights into ongoing and future research activities.

## 2 Methods and Materials

In the event of an unintended human-robot contact, ISO/TS 15066 stipulates that humans must not suffer forms of biomechanical stress beyond the onset of pain. This limitation also applies to the scope of the study presented here. Since there are multiple components of a physical contact, ISO/TS 15066 distinguishes the limits according to the *load type* (i.e., profile of the contact force) and *contact type* (i.e., shape of the contact area). Both features were essential for the methods used in the study as the following describe.

The load type indicates whether the contact is impact or pinching. In the case of an impact, the contact force applied by the robot builds up quickly and decreases again quickly after reaching its maximum. If the contact force builds up slowly, this indicates a pinching contact. The force over time does not show an exposed maximum and remains at a constant value when the robot has stopped. The contact type can either be *semi-sharp* or *blunt*. According to the requirements of ISO/TS 15066, sharp contact shapes are not allowed on the surface of a cobot. Behrens and Elkmann (2021) identified all shapes with an effective contact area of 0.5 cm^2^ as sharp.

Otto von Guericke University Magdeburg’s ethics committee approved the study (reference numbers 37/15 and 13/19). All subjects were insured against injuries that could possibly result from the load tests executed during the study. Written informed consent was obtained from the individuals for the publication of any potentially identifiable images included in this article.

### 2.1 Work Plan and Procedure

**FIGURE 1:**
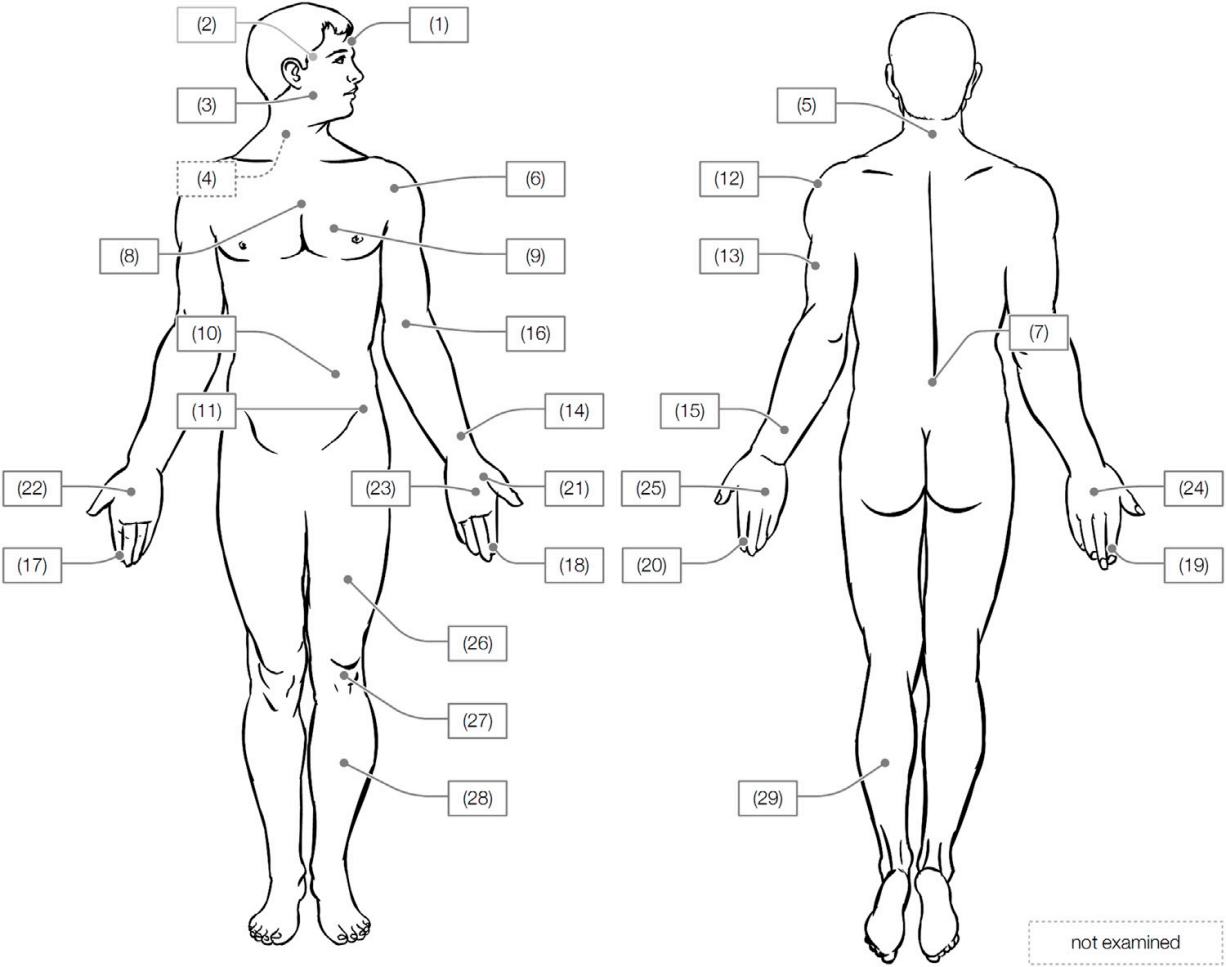
Tested body locations.

Table 1 presents the study’s work plan, which includes the body locations tested, the test parameters varied, and the values recorded. Figure 1 depicts the exact positions of all body locations and their identification numbers (names of the body locations can be found in Table 2). Each body location was precisely pinpointed by means of a localizing procedure that uses various anatomical landmarks of the body to ensure the load was always applied to exactly the same body location in consecutive tests.

**TABLE 1:** Work plan including all test parameters and conditions.

Subject group	G1	G2	G3	G4		G5
Body locations (see Figure 1)	(6)	(6)	(6)	(1)	(1)	(1)
	⋮	(7)	(7)	⋮	⋮	⋮
	(29)	(11)	(11)	(3)	(3)	(3)
		⋮	⋮	(5)	(5)	(5)
		(29)	(29)		(8)	⋮
					⋮	(29)
			OND^a^		(10)	
Load type						
Pinching	*✓*			*✓*		*✓*
Impact		*✓*	*✓*		*✓*	
Contact type (see Figure 4)						
Semi-sharp (contact body F-Q10)		*✓*	*✓*		*✓*	*✓*
Blunt (contact body F-C30)	*✓*	*✓*	*✓*	*✓*	*✓*	
Repeats	5	2^b^	2^b^	3	2^b^	3
Quantities measured						
Force	*✓*	*✓*	*✓*	*✓*	*✓*	*✓*
Tissue deformation	*✓*	*✓*	*✓*	*✓*	*✓*	*✓*
Pressure		*✓* ^c^	*✓* ^c^		*✓*	*✓*

a only the non-dominant hand.

b both tests with different impact masses (see Section 2.2.2).

c only for tests with F-Q10.

**TABLE 2:** Assignment of the subject groups to the experiments.

Body part	Body location	Pinching		Impact	
		Semi-sharp	Blunt	Semi-sharp	Blunt
Head and neck	(1) Forehead	G5	G4	G4	G4
	(2) Temple	G5	G4	G4	G4
	(3) Masticatory m.	G5	G4	G4	G4
	(4) Neck m.	—	—	—	—
	(5) 7th cervical v. (C7)	G5	G4	G4	G4
Trunk	(6) Shoulder joint	G5	G1	G2/3	G2/3
	(7) 5th lumbar v. (L5)	G5	G1	G2/3	G2/3
	(8) Sternum	G5	G1	G4	G4
	(9) Pectoral m.	G5	G1	G4	G4
	(10) Abdominal m.	G5	G1	G4	G4
	(11) Pelvic b.	G5	G1	G2/3	G2/3
Upper extremities	(12) Deltoid m.	G5	G1	G2/3	G2/3
	(13) Humerus	G5	G1	G2/3	G2/3
	(14) Radial b.	G5	G1	G2/3	G2/3
	(15) Forearm m.	G5	G1	G2/3	G2/3
	(16) Arm nerve	G5	G1	G2/3	G2/3
Hand and fingers	(17) Forefinger pad D	G5	G1	G2	G2
	(18) Forefinger pad ND	G5	G1	G2/3	G2/3
	(19) Forefinger DIP D	G5	G1	G2	G2
	(20) Forefinger DIP ND	G5	G1	G2/3	G2/3
	(21) Thenar eminence ND	G5	G1	G2/3	G2/3
	(22) Palm D	G5	G1	G2	G2
	(23) Palm ND	G5	G1	G2/3	G2/3
	(24) Back of the hand D	G5	G1	G2	G2
	(25) Back of the hand ND	G5	G1	G2/3	G2/3
Lower extremities	(26) Thigh m.	G5	G1	G2/3	G2/3
	(27) Kneecap	G5	G1	G2/3	G2/3
	(28) Middle of shin	G5	G1	G2/3	G2/3
	(29) Calf m.	G5	G1	G2/3	G2/3

m, muscle; v, vertebra; b, bone; D, dominant hand; ND, non-dominant hand; DIP, distal interphalangeal (end joint).

The risk of injury to the subjects posed by the load tests was assessed medically prior to the study. One important outcome of the assessment was the decision to exclude load tests on the neck muscle that lies close to nerve tracts for essential bodily functions. The physicians involved in our study concluded that especially impact loads applied to this region could compromise the nerve tracts’ function with unpredictable consequences for the subject. Moreover, the physicians recommended to examine the body locations in the order given by Table 1. Initial tests on body locations with high natural resistance to external loads (i.e., upper and lower extremities) have demonstrated that the forces to evoke pain are significantly lower than those that can cause injury. Only after we confirmed this assumption was it acceptable to proceed with the tests on other body locations with lower resistance (e.g., abdominal muscle). Body location (9) pectoral muscle was eliminated from tests with female subjects out of respect for personal boundaries.

The experimental procedure varied depending which of the two testing systems was used (see Sections 2.2.1, 2.2.2). The preparation of each test was, however, identical for both systems. First, the experimenter configured the test parameters according to the session’s work plan. Here, a session denotes a series of individual tests, usually performed with one subject on 1 day. Then, the subject was placed in the testing system and the body part was secured. To be consistent with the procedure followed by Melia et al. (2019) and Park et al. (2019), the subject was reminded to stop the load test the moment the pressure felt at the body location changed to a slight painful feeling. In each subject’s first session, several test runs were executed to familiarize them with the procedure and to train their ability to distinguish the pain from discomfort.

### 2.2 Experimental Setup

The human body responds differently to impact and pinching loads. Thus, investigating the human pain threshold for both contact types requires appropriate testing systems. In our study, we decided to utilize an algometer to emulate pinching loads and a pendulum to emulate impact loads, as described in the following.

#### 2.2.1 Algometer

**FIGURE 2:**
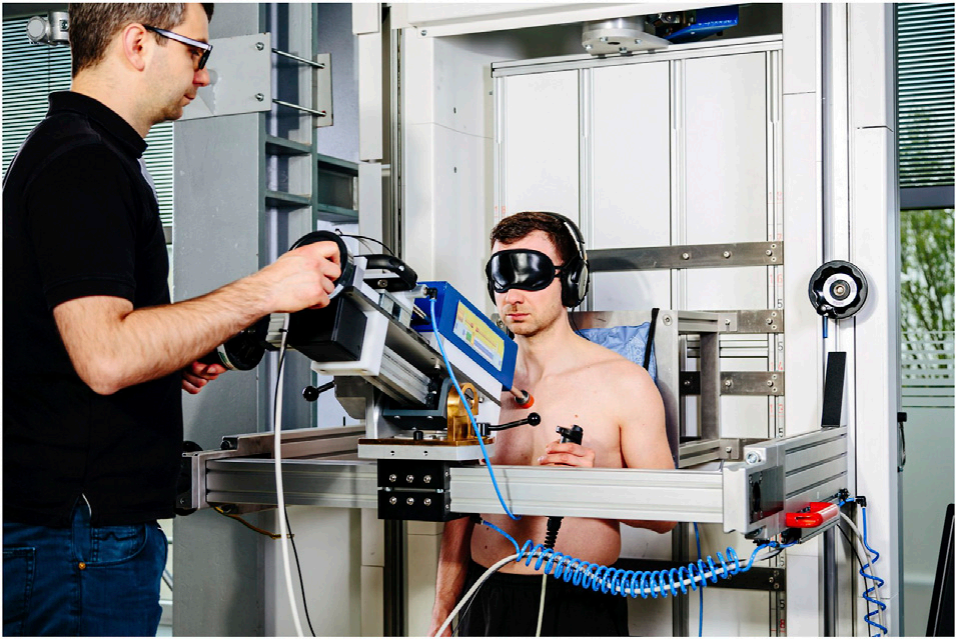
Algometer used to determine limits for pinching contacts.

The algometer (see Figure 2) resembles the design of the one utilized in other studies for similar purposes (Yamada et al., 1996; Saito and Ikeda, 2005; Melia et al., 2014; Melia et al., 2019). Its main component is a manually operated mechanism that pushes the contact body against the test subject’s body location. Multiple linear guides and joints enable the operator to position the loading mechanism perpendicular to any region of the body. A hand switch activates the force transmission between the manual drive (realized by a hand crank) and the rod to which the contact body is affixed. During a load test, the subject pressed a hand switch to its second position to enable force transmission from the drive to the rod. After the subject enabled the transmission, the operator began to increase the pinching force by turning the crank, which then pushes the rod with the contact body on top forward. The deformation rate was 1 mm/s as used in the study of Park et al. (2019). As soon as the subject felt a slight pain at the loaded body location, they were instructed to press the switch to the last position, whereupon the rod immediately recoiled and the load ceased. Every single test on a single body location was performed at least three times (see Table 1). The idle time between two tests was at least 45 s and thus sufficient to prevent an influence of the repeats on the subjects’ pain thresholds (Brennum et al., 1989; Defrin et al., 2003).

A single-axis load cell inside the loading mechanism recorded the contact force (KISTLER 9311B; range ±500 N; max. error 2.11%). The state of the enabling switch was also recorded to precisely determine the force that evoked the painful feeling. An inductive encoder (Micro-Epsilon VIP150; range 150 mm, max. error 1.24%) supported the operator to maintain the deformation rate. All signals were sampled by an A/D converter (Meilhaus ME-4660i PCIE) at 100 Hz and 16 bit. The sampling frequency is the same as used in the studies of Melia et al. (2019) and Park et al. (2019).

#### 2.2.2 Pendulum

**FIGURE 3:**
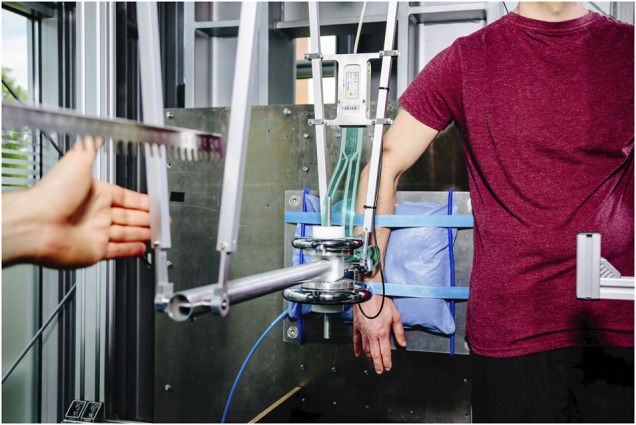
Pendulum used to determine limits for impacts.

The pendulum (see Figure 3) resembles a four-bar linkage with two parallel bars, each measuring 0.8 m in length. The bars connect the pendulum body to multiple linear guides that enable the operator to adjust the pendulum’s position to any body regions. The pendulum body has an interface to increase its net weight from 1.9 to 20 kg. A lever with equidistant notches on the back locks the pendulum in starting position. The maximum impact velocity is 1.25 m/s and can be adjusted in increments of approximately 0.01 m/s.

For executing an impact test, the operator first deflected the pendulum body to the starting position and released it after the instruments began taking measurements. When the pendulum recoiled after striking the subject, it was caught at the rear bars and deflected to the next higher position. This procedure was repeated multiple times until the subject verbally informed the experimenter that the last impact had caused pain.

From a simplified impact model, we identified how to change the impact velocity *v*_*P*_ and pendulum mass *m*_*P*_ over the repeats. The model assumes that the human tissue under load has linear elasticity *c*_*H*_ and zero viscosity. Then, the maximum contact force ${\hat{F}}_{C}$ is approximately proportional to *v*_*P*_ (Hodgson et al., 1965; Haddadin et al., 2009)

$${\hat{F}}_{C}\approx \sqrt{m_{P}c_{H}}v_{P}.$$ (1)

According to the model, it seemed to be convenient to increase *v*_*P*_ proportional in fixed steps of 0.05 m/s, except for tests on the head, where the step size was reduced to 0.01 m/s as a precaution. It must be noted that the model is a considerable simplification. In fact, the hyper-elasticity of soft tissue paired with viscosity determines its response under impact and pinching load (Fung, 1993). The work of other researchers, however, has confirmed that, firstly, the model gives a good estimation of ${\hat{F}}_{C}$ and, secondly, includes all relevant parameters with significantly influence on an impact’s intensity (Hodgson et al., 1965; Haddadin et al., 2009).

The waiting time between two subsequent impacts was approximately 5 s. Given the findings from other studies, there is no clear evidence that multiple loads applied in such short order affect a subject’s pain threshold (Brennum et al., 1989; Kosek et al., 1993; Isselée et al., 1997; Chesterton et al., 2003). To take also into account the effect of impact mass on ${\hat{F}}_{C}$ , we performed the first tests with a pendulum weight of 16.5 kg and the second with a pendulum weight of 6.5 kg. Both masses are approximately in the range of the apparent mass that a cobot has during an impact (Haddadin et al., 2009; Haddadin et al., 2012).

The interface to affix the contact body at the pendulum’s body front is part of the piezoelectric load cell (KISTLER 9327C; range ±1 kN, max. error 1.61%) that records the contact force in all three directions in space. The same A/D converter as used for the algometer sampled the signals at 10 kHz and 16 bit. Sampling frequencies at this level are often used in impact studies, such as that of Dhaliwal et al. (2002) or Povse et al. (2011).

#### 2.2.3 Contact Bodies

**FIGURE 4:**
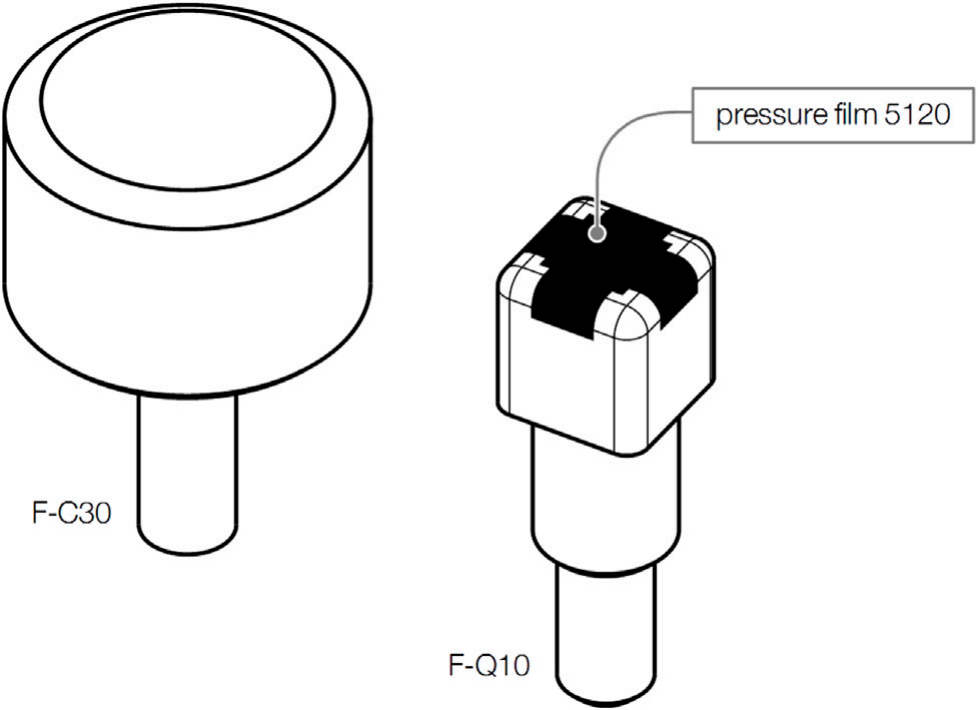
Contact bodies to emulate blunt (F-C30) and semi-sharp (F-Q10) contacts.

Since the review of related studies failed to uncover contact bodies that are perfectly suited for testing semi-sharp and blunt contacts, this study employed a semi-sharp contact body, F-Q10 (see Figure 4), identical to the one used by Melia et al. (2014), Melia et al. (2019) and Park et al. (2019). It is a cuboid made of aluminum with a rectangular area measuring 14 mm × 14 mm. Edges and corners were all rounded to a radius of 2 mm. The cross-section area of F-Q10 is 1.96 cm^2^ and thus beyond the region of sharp contact bodies (Behrens and Elkmann, 2021). A piezoelectric film (TekScan I-Scan, type no 5120, range 1.2 kN/cm^2^, max. error ≤10%) was attached to the face of F-Q10 for pressure measuring. The signal converter for the films sampled the pressure at 2.07 kHz and 8 bit.

Contact body F-C30 was developed to emulate blunt contacts (see Figure 4). Its circular contact surface with a diameter of 30 mm is significantly larger than that of F-Q10. Initial tests have demonstrated that contact body of such a diameter can be applied to all 28 body locations without overlapping to other body locations. F-C30 is made of a compliant foam that prevents distinct pressure regions in the contact area. The foam has an average elasticity of 24.4 MPa/m. A pressure film was not affixed to F-C30.

#### 2.2.4 Additional Provisions

During the tests, the subject’s body part was affixed to a socket held by a rigid frame. Vacuum cushions and straps kept the body part from slipping or shifting. Various positioning aids helped to align the body locations under test precisely with the force-inducing part of the testing system used. The subjects stood in an upright and relaxed position most of the time. They wore sleep masks and headphones playing nature sounds. Both actions were taken to isolate the subjects from their surroundings and prevented the subjects from instinctively tensing their muscles right before the load was applied. Only a few body locations, e.g. on the head or chest, required the subjects to sit or lean slightly forward in a standing position.

### 2.3 Data Processing

The force signal recorded in a load test is $f_{C}\left(t\right)\in R^{N\times 1}$ , where *N* = 1 applies for signals taken with the algometer and *N* = 3 for signals taken with the pendulum. In the tests with contact body F-Q10, the pressure film recorded the pressure (i.e., normal stress) over time *t* at discrete locations *x*_*n*,*m*_ on its sensitive area. The signal can be, therefore, interpreted as a time-dependent stress field ***ψ***_*C*_(*x*, *t*). Before the maximum values from both signals could be extracted, various disturbances that impaired the signals’ quality had to be compensated. This was done with a fully automated MATLAB script which processed the signals as follows.

#### 2.3.1 Filtering and Offset Elimination

A phase-zero and fourth-order Butterworth low-pass filter was applied to reduce high-frequency noise in the signals. The filter parameters selected comply with the channel frequency class (CFC) specified in (J211-1 S, 2014). A Fast Fourier Transform (FFT) analysis of the signals from the algometer showed that a CFC1 filter suffices to reduce the noise. A CFC100 filter was determined to be necessary for the signals acquired with the pendulum. The offsets in the force signals were also eliminated so that the contact force was exactly zero at the moment of initial contact.

A blur filter was required to clean the image-based pressure signals from image noise. The sensor’s manufacturer recommends applying a Gaussian blur filter with a 3 × 3 kernel and a variance of *σ* = 2/*π*. Since the sensor film was bent slightly over the edges of F-Q10 (see Figure 4), it was necessary to separately blur the pixels in the images that appear in one of the sensor’s five views (one overhead view and four lateral views). Since the gray-scale values of an image’s pixels exhibit magnitudes of stress vectors, the filter algorithm reduced them to the fractions the sensor can record in the direction of the normal vector of the view being filtered. The separately blurred views were then reassembled to one image.

#### 2.3.2 Pressure Interpolation

**FIGURE 5:**
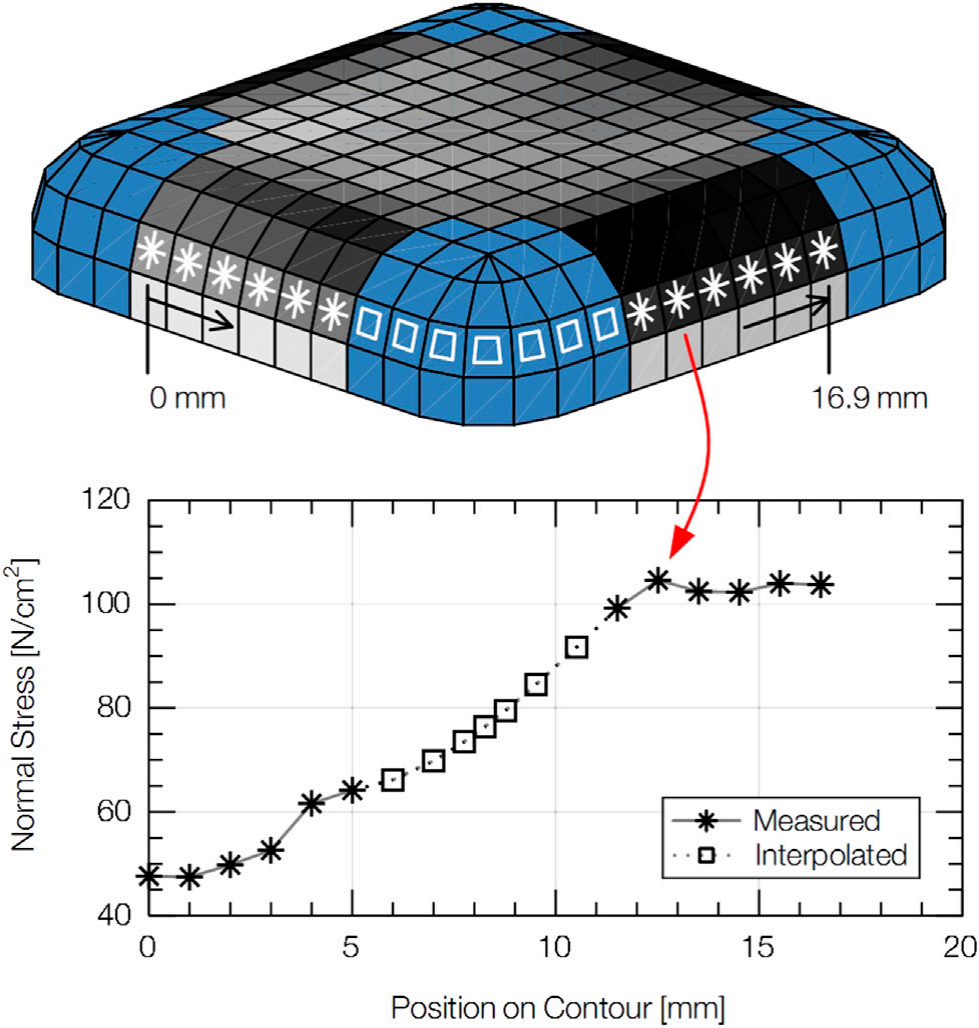
Technique used to interpolate pressure values in the blind spots of the pressure film affixed to F-Q10.

The regions next to the dark area on the illustration of F-Q10 in Figure 4 show that the pressure film did not cover its entire face. An interpolation was, therefore, needed to fill the blind spots with estimates. As Figure 5 shows, the applied technique first extracted all values from the pressure image, which lie on the same outer contour of F-Q10. Mapping these values over their positions along the contact body’s outer contour gives a curve with gaps, which were closed by spline interpolation. Applying the method to all contours ultimately filled the blind spots with estimates. A scaling operation at the end of the interpolation assured that the particular pressure values sum up to the corresponding contact force that was measured at the same time as the pressure image was taken. Melia et al. (2019) present a similar approach for the same contact body, but based on linear interpolation.

#### 2.3.3 Inertia Compensation

Given the distribution of the pendulum mass, the force **f**_*M*_(*t*) measured by the load cell deviates from the contact force **f**_*C*_(*t*) that acted at the tip of the contact body (Nahum et al., 1972; Stalnaker and Melvin, 1976). The total mass of the pendulum acting on the subject consists of the mass of the pendulum body *m*_*B*_ and the mass of the contact body *m*_*I*_, but during an impact only *m*_*B*_ acts on the sensor placed between *m*_*B*_ and *m*_*I*_. Because of the mass constellation, **f**_*C*_ must be multiplied by the factor *V*_**f**_

$$f_{C}\left(t\right)=V_{f}f_{M}\left(t\right)$$ (2)

which is given by

$$V_{f}=1+\frac{m_{I}}{m_{B}}.$$ (3)

#### 2.3.4 Relevant Values

In order to comply with the current structure of ISO/TS 15066, the limits must be based on the *maximum contact force* and *peak pressure*. The maximum contact force ${\hat{F}}_{C}$ is defined as the highest magnitude of the normalized force signal

$${\hat{F}}_{C}=\max_{t}\left\|f_{C}\left(t\right)\right\|.$$ (4)

The peak pressure ${\hat{\psi }}_{C}$ resembles the highest concentration of force measured in the sensitive area of the pressure film on contact body F-Q10. It can be easily determined from ***ψ***_*C*_(**x**, *t*) by the following operation

$${\hat{\psi }}_{C}=\underset{t,x}{\max}\psi_{C}\left(x,t\right).$$ (5)

### 2.4 Subjects

Only female and male participants of working age, i.e. between 16 and 67 in Germany, were considered for the study. All participants were randomly selected from the group of suitable candidates. The medical assessment of each candidate’s health excluded all of them with preexisting conditions that could have either caused complications or biased the results.

Since funding was only available on an irregular basis, the study had to be split into five separate, consecutive phases. Every phase included experiments with one of five groups. Table 3 presents the group's body parameters. Table 1 breaks down how the composition of the groups and the parameters applied in the tests altered over the study’s phases based on experience from previous phases and an adjustment of the requirements specified by the funding body. For instance, the larger number of males in groups G1 and G2 traces back to the initial condition that at least 30% of the subjects had to be blue-collar workers as well as the impossibility of recruiting just as many female workers as male workers within the given preparation time. From the third phase on, the condition was rejected, because of two reasons. First, the requirement that the groups must consist of equally distributed genders was given the highest priority. Second, the participation of blue-collar workers did not affect the results as expected. The choice of body locations to be tested was also corrected. Since it is impossible to know which of a worker’s hands is the dominant one in a working situation with a cobot, testing on this side was rejected for G3. Group G5 served as a control group and was tested in the same way as Melia et al. (2019) and Park et al. (2019) have tested their subjects. The data from G5 were primarily acquired to compare to the data from these studies. Table 2 breaks down the subject groups by test condition, specifically load and contact type.

**TABLE 3:** Body parameters of the subjects.

Group	Females	Males	Both	Age (y)	Height (m)	Weight (kg)
G1	13	28	41	40.6 ± 14.0	1.77 ± 0.10	79.6 ± 16.4
G2	6	14	20	41.9 ± 14.5	1.77 ± 0.08	78.5 ± 14.9
G3	10	10	20	40.2 ± 13.7	1.74 ± 0.08	80.5 ± 17.0
G2/3	16	24	40	41.0 ± 13.9	1.76 ± 0.08	79.5 ± 15.8
G4	10	10	20	39.0 ± 14.0	1.74 ± 0.10	73.8 ± 17.7
G5	5	6	11	36.3 ± 13.4	1.74 ± 0.08	69.1 ± 11.3

## 3 Results

From all tests, we obtained approximately 29 000 individual observations organized in samples. Each sample contains *N* observations from tests with a specific load type *LT* and contact type *CT* applied to one body location *BID* ∈ {1, 2, …, 29} (see Figure 1). Then, a particular observation *y*_*ij*_ can be expressed as a function of *BID*, *LT*, and *CT*

$$y_{ij}:=y_{ij}\left(BID,CT,LT\right).$$ (6)

The indices *i* and *j* relate *y*_*ij*_ to the pain threshold of subject *i* from test *j*. It can either be a maximum contact force or peak pressure. The variable ${\bar{y}}_{i}$ represents the mean of the observations from all repeated tests with subject *i*, whereupon the same functional relationship as in Eq. 6 applies here as well. Note that the repeats of the impact tests were performed with different pendulum masses *m*_*P*_ (see Table 1). These observations will be, however, not separated by mass when processing them to limits. Mass as a third feature to differentiate the limits (in addition to load type and contact type) would increase the complexity of the limit and thus complicate their transfer to ISO/TS 15066.

The samples from the tests are analyzed in the following with commonly used statistical methods. In the first part, we take a look at the empirically distributed observations using descriptive statistics. We then introduce a statistical distribution model to fit the empirical data. Next, the model will be extended with a covariate to account for the effect of gender so that the limits predicted by the model can be precisely tailored to a group of unequally distributed genders.

### 3.1 Descriptive Statistics

The step-wise procedure of approaching the subjects’ pain thresholds with the pendulum resulted in censored observations, which are typical for such studies (Kent et al., 2004). The true but unknown value of the desired observation *y*_*ij*_ lies somewhere within the interval spanned by the results of the last two tests. An estimate of *y*_*ij*_ can be calculated using midpoint imputation, which is a technique that considers *y*_*ij*_ as the mean of the interval boundaries *L*_*ij*_ and *R*_*ij*_ with *L*_*ij*_ < *y*_*ij*_ < *R*_*ij*_ (Sun, 2006). The left boundary *L*_*ij*_ is the value measured in the penultimate test that does not cause any pain. The right boundary *R*_*ij*_ is the value from the last test that causes pain. In the unlikely but possible event that the very first test causes pain, the observation must be treated as fully left-censored with *L*_*ij*_ = 0. Because of the possibility that left-censored observations can occur, it is not recommended to calculate the limits from *L*_*ij*_ and to attenuate them unnecessarily in this way. A second exceptional event occurs when not even the highest impact velocity (approximately 1.25 m/s) causes the subject any pain. Then, *y*_*ij*_ is right-censored with *R*_*ij*_ → *∞*. The following combines both exceptions with the common interval-censored case to one expression

$$y_{ij}\approx \left\{\begin{array}{cc} R_{ij}/2\; & L_{ij}=0\\ L_{ij}\; & R_{ij}\to \infty \\ \left(L_{ij}+R_{ij}\right)/2\; & \text{otherwise}\;. \end{array}\right.$$ (7)

Table 4 presents the arithmetic mean $\bar{y}$ and standard deviation *s* of the averaged observations ${\bar{y}}_{i}$ . The data are sorted by body part, load type and contact type. In addition to the data from the regular subject groups, the table also includes the data from control group G5 (see Table 2). The data of G5 will later compared to other studies with a similar scope and experimental design (see Section 4.5). Note that all values in Table 4 for impact were converted with Eq. 7 before calculating ${\bar{y}}_{i}$ . The data from the algometer tests are not censored and did not require any conversion.

**TABLE 4:** Mean values $\bar{y}$ and standard deviations *s* for the acquired samples consisting of *N* averaged observations.

Body part	Pinching				Impact			
	Semi-sharp		Blunt		Semi-sharp		Blunt	
	$\bar{y}\pm s$ ^a^	*N*	$\bar{y}\pm s$ ^b^	*N*	$\bar{y}\pm s$ ^a^	*N*	$\bar{y}\pm s$ ^b^	*N*
(1) Forehead	82 ± 57	11	77 ± 58	19	152 ± 114	20	117 ± 98	20
(2) Temple	43 ± 27	11	48 ± 46	19	62 ± 42	20	72 ± 44	20
(3) Masticatory m.	31 ± 20	11	32 ± 24	18	45 ± 27	20	56 ± 28	20
(5) C7	59 ± 25	9	40 ± 32	18	145 ± 88	20	62 ± 31	20
(6) Shoulder joint	57 ± 51	11	57 ± 25	40	90 ± 34	39	87 ± 35	40
(7) L5	53 ± 46	10	92 ± 66	40	159 ± 62	22	149 ± 76	38
(8) Sternum	44 ± 29	11	65 ± 34	40	63 ± 29	20	91 ± 61	20
(9) Pectoral m.	44 ± 21	6	50 ± 22	27	62 ± 24	10	94 ± 49	10
(10) Abdominal m.	34 ± 27	11	43 ± 28	40	50 ± 25	20	65 ± 38	20
(11) Pelvic bone	107 ± 101	11	77 ± 45	40	224 ± 124	40	128 ± 58	40
(12) Deltoid m.	66 ± 54	11	85 ± 60	40	93 ± 33	39	100 ± 45	40
(13) Humerus	43 ± 32	11	59 ± 30	40	117 ± 60	39	127 ± 44	39
(14) Radial bone	64 ± 53	11	82 ± 41	40	143 ± 59	40	155 ± 70	40
(15) Forearm m.	61 ± 78	11	83 ± 45	40	118 ± 45	40	146 ± 57	39
(16) Arm nerve	50 ± 39	11	63 ± 30	40	112 ± 40	40	126 ± 55	40
(17/18) Forefinger pad	59 ± 39	11	123 ± 84	41	197 ± 88	40	306 ± 160	40
(19/20) Forefinger DIP	117 ± 72	11	126 ± 75	40	424 ± 140	40	310 ± 151	40
(21) Thenar eminence	47 ± 25	11	100 ± 62	40	149 ± 69	40	223 ± 115	40
(22/23) Palm	56 ± 38	11	118 ± 77	40	241 ± 132	40	283 ± 143	40
(24/25) Back of the hand	145 ± 89	11	114 ± 50	40	391 ± 197	40	209 ± 96	40
(26) Thigh muscle	65 ± 46	11	119 ± 62	39	136 ± 50	40	178 ± 85	40
(27) Kneecap	103 ± 79	11	129 ± 73	39	224 ± 99	39	226 ± 117	39
(28) Middle of shin	123 ± 93	11	128 ± 61	40	319 ± 132	40	219 ± 107	40
(29) Calf m.	63 ± 46	11	112 ± 61	40	149 ± 68	40	217 ± 98	40

a pressure values in unit [N/cm^2^].

b force values in unit [N].

### 3.2 Distribution Model

A suitable model to predict the quantile 0 < *q* < 1 for an arbitrary observation *y* can be created using a cumulative distribution function (CDF) F:y→q . To determine the desired limits, we need the inversion of F(y) , which predicts the observation *y*_*q*_ for a given *q*

$$y_{q}=F^{-1}\left(q\right).$$ (8)

**FIGURE 6:**
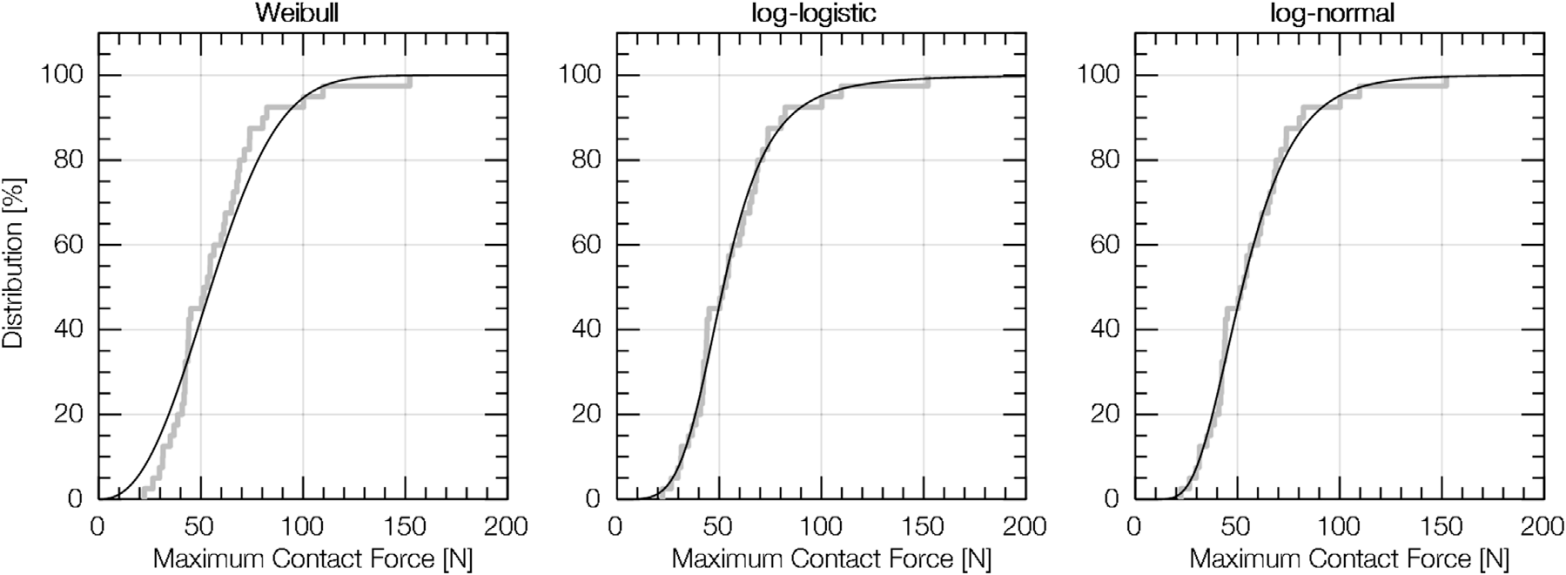
Empirical distribution (EDF) and cumulative distribution function (CDF) for (12) deltoid muscle (blunt pinching, i.e. data from tests with the algometer and F-C30).

In traumatology and injury biomechanics, frequently used CDFs base on the Weibull, log-logistic and log-normal distribution model (Kent et al., 2004). An Anderson Darling test applied to our data revealed that the log-logistic CDF has the best fit to reproduce the empirical distribution (EDF) of most samples’ observations (see Figure 6). The log-logistic CDF is given by

$$F\left(y\right)={\left(1+\exp\left[-\frac{\log y-\beta_{0}}{\alpha }\right]\right)}^{-1}\;,$$ (9)

where *β*_0_ is the scale and *α* the shape parameter. According to multiple studies (Fischer, 1986; Buchanan and Midgley, 1987; Fischer, 1987; Brennum et al., 1989; Takala, 1990; Hogeweg et al., 1992; Lee et al., 1994; Fillingim and Maixner, 1995; Fredriksson et al., 2000), the gender was confirmed to have an effect on the pain thresholds’ tendency. Other covariates such as BMI or age are not or less significant (see articles mentioned). In this light and from a statistical view point, it appears reasonable to incorporate the gender as a covariate into Eq. 9, which can be achieved using the Accelerated Failure Time (AFT) model. It uses the following Weibull regression (Lawless and Lawless, 2003)

$$V\left(x\right)=\exp\left[x^{T}\beta \right]$$ (10)

to reproduce fixed effects ***β*** of the covariates **x**, by simply shifting the unbiased basic quantile *y* ^*B*^ to the specific value *y*

$$y=V\left(x\right)y^{B}.$$ (11)

Solving Eq. 11 for *y* ^*B*^ and using it as an substitute for the argument of Eq. 9 finally expands Eq. 9 as desired

$$F\left(y^{B}\right)=F\left(y,x\right).$$ (12)

If the gender is considered as the only covariate, **x** is scalar and resembles *x*_*G*_ with 0 ≤ *x*_*G*_ ≤ 1, where *x*_*G*_ = 0 corresponds to a wholly female group and *x*_*G*_ = 1 to a wholly male group. Everything in between expresses a mixed-gender group. With *x*_*G*_ being the only covariate, solving Eq. 12 finally gives the expanded model

$$F\left(y,x_{G}\right)={\left(1+\exp\left[-\frac{\log y-\beta_{0}-x_{G}\beta_{1}}{\alpha }\right]\right)}^{-1}$$ (13)

We used the statistical software **R** and the survival package to estimate the intercept *β*_0_, effect of the gender *β*_1_, and shape parameter *α* for every sample. Except the parameters, the estimation has also calculated the *p* values that indicate the significance of covariate *x*_*G*_ (for significance level *σ* = 0.05). The null-hypothesis *H*_0_ tested here presumes that *β*_1_ is zero, meaning the gender has no effect.

The *p* values obtained are listed in Table 5. Altogether, there are 45 cases where *H*_0_ was rejected (gender is likely to have an effect). Given this result, we consider *x*_*G*_ as relevant if the *p* values to a body location are below *σ* for at least two experimental conditions (out of four possible). Otherwise, we can assume *β*_1_ = 0 so that we can keep the structure of Eq. 13. A less general approach that only assumes *β*_1_ > 0 whenever *H*_0_ was rejected did not seem appropriate because there is, to our best knowledge, no scientific evidence that the conditions of a physical contact affect the gender difference.

**TABLE 5:** *p* values exhibiting the significance of the covariate *x*_*G*_ that integrates the gender’s effect on the pain thresholds in the extended distribution model (significance level *σ* = 0.05); coefficients of determination *R* ^2^ indicating the fitting quality of the distribution model.

Body part	Pinching				Impact			
	Semi-sharp		Blunt		Semi-sharp		Blunt	
	*p*	*R* ^2^	*p*	*R* ^2^	*p*	*R* ^2^	*p*	*R* ^2^
(1) Forehead	0.098	0.94	0.455	0.94	0.098	0.98	0.214	0.97
(2) Temple	0.450	0.94	0.302	0.97	0.282	0.95	0.436	0.98
(3) Masticatory m.	0.228	0.91	0.406	0.97	0.262	0.98	0.476	0.97
(5) C7	0.122	0.96	0.237	0.97	0.007	0.90	0.050	0.98
(6) Shoulder joint	0.276	0.95	0.083	0.98	0.017	0.98	0.020	0.98
(7) L5	0.218	0.97	0.127	0.98	0.295	0.95	0.003	0.98
(8) Sternum	0.077	0.97	<0.001	0.97	<0.001	0.89	0.019	0.91
(9) Pectoral m.	<0.001	0.84	0.806	0.97	<0.001	0.93	<0.001	0.95
(10) Abdominal m.	<0.001	0.85	0.005	0.97	0.083	0.96	0.010	0.97
(11) Pelvic bone	0.033	0.78	0.044	0.99	0.342	0.99	<0.001	0.96
(12) Deltoid m.	0.067	0.94	0.027	0.95	<0.001	0.96	<0.001	0.97
(13) Humerus	0.044	0.84	0.016	0.98	<0.001	0.96	<0.001	0.98
(14) Radial bone	0.111	0.97	0.006	0.97	0.017	0.99	0.003	0.98
(15) Forearm m.	0.012	0.95	0.104	0.98	0.005	0.99	0.058	0.98
(16) Arm nerve	0.414	0.92	0.155	0.99	0.450	0.98	0.403	0.99
(17/18) Forefinger pad	0.113	0.94	0.476	0.98	0.220	0.99	0.347	0.98
(19/20) Forefinger DIP	0.168	0.96	0.389	0.99	0.514	0.99	0.192	0.99
(21) Thenar eminence	0.365	0.93	0.102	0.98	0.956	0.99	0.240	0.99
(22/23) Palm	0.003	0.84	0.016	0.97	0.040	0.98	0.067	0.98
(24/25) Back of the hand	0.489	0.94	0.716	0.99	0.095	0.99	0.045	0.99
(26) Thigh muscle	0.053	0.94	0.032	0.97	0.022	0.98	<0.001	0.98
(27) Kneecap	0.008	0.93	0.160	0.98	<0.001	0.92	0.007	0.97
(28) Middle of shin	0.004	0.91	0.251	0.98	<0.001	0.97	0.025	0.97
(29) Calf m.	0.151	0.97	0.009	0.98	<0.001	0.97	0.002	0.96

The parameters estimated for the distribution model Eq. 13 can be found in Table 6. The coefficients of determination *R* ^2^, which illustrate the model’s fitting quality, are listed in Table 5. According to these values, model Eq. 13 fit the empirical observations well, both for *β*_1_ > 0 and *β*_1_ = 0.

**TABLE 6:** Estimated parameters of the expanded distribution model based on the log-logistic cumulative distribution function (CDF) and Weibull regression; *β*_0_ is the intercept, *β*_1_ the effect of gender, and *α* the shape parameter of the CDF.

Body part	Pinching						Impact					
	Semi-sharp			Blunt			Semi-sharp			Blunt		
	*β*_0_	*β*_1_	*α*	*β*_0_	*β*_1_	*α*	*β*_0_	*β*_1_	*α*	*β*_0_	*β*_1_	*α*
(1) Forehead	4.22	0	0.46	4.10	0	0.53	4.83	0	0.32	4.50	0	0.42
(2) Temple	3.59	0	0.37	3.50	0	0.56	3.96	0	0.32	4.12	0	0.35
(3) Masticatory m.	3.26	0	0.30	3.16	0	0.49	3.67	0	0.26	3.90	0	0.29
(5) C7	4.02	0	0.25	3.44	0	0.43	4.80	0	0.33	4.01	0	0.27
(6) Shoulder joint	3.54	0.38	0.35	3.81	0.22	0.21	4.29	0.24	0.18	4.21	0.30	0.23
(7) L5	3.65	0	0.47	4.31	0	0.36	5.01	0	0.24	4.90	0	0.29
(8) Sternum	3.26	0.63	0.34	3.61	0.64	0.30	3.72	0.63	0.21	4.02	0.55	0.29
(9) Pectoral m.	3.67	0.00	0.27	3.74	0.09	0.25	4.07	0.00	0.22	4.42	0.00	0.29
(10) Abdominal m.	2.89	0.84	0.24	3.21	0.55	0.34	3.59	0.32	0.24	3.71	0.54	0.27
(11) Pelvic bone	3.81	1.15	0.53	3.96	0.36	0.30	5.16	0.18	0.32	4.44	0.51	0.21
(12) Deltoid m.	3.57	0.63	0.35	3.96	0.40	0.32	4.24	0.37	0.15	4.19	0.52	0.23
(13) Humerus	3.25	0.57	0.27	3.72	0.36	0.25	4.35	0.45	0.25	4.56	0.38	0.19
(14) Radial bone	3.56	0.60	0.37	4.05	0.39	0.24	4.71	0.28	0.21	4.74	0.37	0.22
(15) Forearm m.	3.11	0.90	0.36	4.14	0.25	0.26	4.54	0.28	0.18	4.77	0.22	0.20
(16) Arm nerve	3.62	0	0.41	4.05	0	0.29	4.65	0	0.18	4.74	0	0.22
(17/18) Forefinger pad	3.90	0	0.42	4.61	0	0.37	5.20	0	0.25	5.60	0	0.33
(19/20) Forefinger DIP	4.62	0	0.32	4.67	0	0.35	6.00	0	0.19	5.62	0	0.28
(21) Thenar eminence	3.74	0	0.32	4.45	0	0.32	4.91	0	0.26	5.28	0	0.27
(22/23) Palm	3.47	0.71	0.24	4.30	0.47	0.34	5.20	0.28	0.25	5.36	0.27	0.26
(24/25) Back of the hand	4.82	0	0.29	4.67	0	0.28	5.86	0	0.31	5.25	0	0.26
(26) Thigh muscle	3.65	0.63	0.32	4.39	0.38	0.29	4.69	0.27	0.21	4.76	0.49	0.24
(27) Kneecap	3.82	0.96	0.37	4.55	0.25	0.32	4.91	0.67	0.21	5.00	0.46	0.29
(28) Middle of shin	3.85	1.17	0.40	4.64	0.15	0.24	5.36	0.51	0.24	5.08	0.35	0.27
(29) Calf m.	3.60	0.56	0.38	4.29	0.44	0.28	4.68	0.42	0.23	5.02	0.44	0.24

### 3.3 Calculation of Limits

With Eq. 8, model Eq. 13, and the parameters from Table 6, we calculated for *q* = 0.75 (75th percentile) the basic quantiles and their confidence intervals (for confidence level 95%) listed in Table 7. Our decision to use *q* = 0.75 traces back to the limits for semi-sharp pinching of ISO/TS 15066, which are associated to the same percentile. For all body locations where gender is presumed to have an effect (*β*_1_ > 0), we assumed a group of workers in which males make up 70% (*x*_*G*_ = 0.7), although the percentage of females working in the European manufacturing sector is lower (Eurostat, 2021).

**TABLE 7:** Basic quantiles *y* ^*B*^ calculated with Eq. 8 for *q* = 0.75; pressure $y=\hat{\psi }$ in [N/cm^2^] and maximum force $y=\hat{F}$ in [N]; confidence interval of *y* ^*B*^ for 95% confidence level is given by the lower boundary ${\hat{y}}^{L}$ and upper boundary ${\hat{y}}^{U}$ ; last column indicates whether the effect of gender on the limits is significant.

Body part	Pinching						Impact							*x*_*G*_
	Semi-sharp			Blunt			Semi-sharp			Blunt				
	${\hat{\psi }}^{L}$	${\hat{\psi }}^{B}$	${\hat{\psi }}^{U}$	${\hat{F}}^{L}$	${\hat{F}}^{B}$	${\hat{F}}^{U}$	${\hat{\psi }}^{L}$	${\hat{\psi }}^{B}$	${\hat{\psi }}^{U}$	${\hat{F}}^{L}$	${\hat{F}}^{B}$		${\hat{F}}^{U}$	
(1) Forehead	57	113	252	59	107	211	125	178	267	90	142		240	No
(2) Temple	32	54	102	32	61	124	53	74	110	62	90		139	No
(3) Masticatory m.	23	36	62	23	40	77	39	52	72	49	68		97	No
(5) C7	49	73	119	30	50	89	121	174	262	55	74		104	No
(6) Shoulder joint	42	62	93	55	63	72	91	102	114	89	102		117	Yes
(7) L5	31	64	151	83	109	149	152	197	263	145	184		239	No
(8) Sternum	33	49	73	66	79	96	68	82	98	81	106		138	Yes
(9) Pectoral m.	34	52	81	45	59	77	57	74	96	80	114		162	Yes
(10) Abdominal m.	37	54	81	48	61	78	49	65	85	69	94		128	Yes
(11) Pelvic bone	80	143	256	78	94	112	225	275	336	124	141		161	Yes
(12) Deltoid m.	48	71	106	80	97	119	94	103	113	100	115		132	Yes
(13) Humerus	33	46	64	60	70	81	114	134	156	132	149		167	Yes
(14) Radial bone	46	71	109	85	98	113	146	166	188	158	181		206	Yes
(15) Forearm m.	34	52	80	85	99	116	121	135	151	147	168		190	Yes
(16) Arm nerve	33	59	115	63	79	102	111	128	151	123	146		176	No
(17/18) Forefinger pad	43	78	155	112	151	207	195	239	297	300	391		522	No
(19/20) Forefinger DIP	88	144	258	119	157	213	427	496	586	301	375		477	No
(21) Thenar eminence	38	60	103	94	121	159	146	179	224	213	265		336	No
(22/23) Palm	45	59	78	119	146	178	240	280	326	282	332		390	Yes
(24/25) Back of the hand	114	171	276	116	145	184	384	488	635	206	252		315	No
(26) Thigh muscle	52	74	106	119	142	171	142	161	182	175	204		237	Yes
(27) Kneecap	72	110	166	130	159	194	226	257	293	224	269		324	Yes
(28) Middle of shin	85	132	205	128	149	172	323	374	433	225	265		312	Yes
(29) Calf m.	48	74	114	113	134	160	155	179	206	222	258		299	Yes

Table 8 shows a proposal how the limits could be arranged in ISO/TS 15066. A comparison of the limits for both load types makes clear that it is reasonable to have separate limits for impact and pinching. The limits for impact clearly exceed those for pinching as expected. The following quotient can be used to exhibit the relative difference

$$w_{q}:=w_{q}\left(BID,CT\right)=\frac{y_{q}\left(BID,LT=\langle \text{impact}\rangle ,CT\right)}{y_{q}\left(BID,LT=\langle \text{pinching}\rangle ,CT\right)},$$ (14)

where *y*_*q*_ is again the quantile function Eq. 8 that calculates limits for a given body location, load type, and contact type. As already mentioned earlier, *y*_*q*_ can either be a force-based limit (for *CT* = ⟨blunt⟩) or a pressure-based limit (for *CT* = ⟨semi-sharp⟩). The average relative difference ${\bar{w}}_{q}\left(CT\right)$ is then the mean calculated from *w*_*q*_ for all body locations. For *q* = 0.75 and *x*_*G*_ = 0.7, we obtain for semi-sharp contacts

$${\bar{w}}_{0.75}\left(CT=\left\langle \text{semi}-\text{sharp}\right\rangle \right)=2.8$$

with *w*_0.75_(*BID*, *CT* = ⟨semi-sharp⟩) ranging from 1.4 to 5.6, and for blunt contacts

$${\bar{w}}_{0.75}\left(CT=\left\langle \text{blunt}\right\rangle \right)=1.8$$

with *w*_0.75_(*BID*, *CT* = ⟨semi-sharp⟩) ranging from 1.1 to 2.7. These results approximately match the trends observed by Yamada et al. (1996).

**TABLE 8:** Proposed limits for safe pHRI; pressure and force limits reflect the pain threshold of the 75th percentile of a group in which males make up 70%.

Body part	Pinching		Impact	
	$\hat{\psi }$ [N/cm^2^]	$\hat{F}$ [N]	$\hat{\psi }$ [N/cm^2^]	$\hat{F}$ [N]
(1) Forehead	110	110	180	150
(2) Temple	50	60	70	90
(3) Masticatory m.	40	40	50	70
(4) Neck m^a^	60	70	80	110
(5) C7	70	50	170	70
(6) Shoulder joint	60	60	100	100
(7) L5	60	110	200	180
(8) Sternum	50	80	80	110
(9) Pectoral m.	50	60	70	110
(10) Abdominal m.	50	60	60	90
(11) Pelvic bone	140	90	270	140
(12) Deltoid m.	70	100	100	110
(13) Humerus	50	70	130	150
(14) Radial bone	70	100	170	180
(15) Forearm m.	50	100	140	170
(16) Arm nerve	60	80	130	140
(17/18) Forefinger pad	70	150	240	390
(19/20) Forefinger DIP	150	160	490	370
(21) Thenar eminence	60	120	180	260
(22/23) Palm	60	150	280	330
(24/25) Back of the hand	170	150	470	250
(26) Thigh muscle	70	140	160	200
(27) Kneecap	110	160	260	270
(28) Middle of shin	130	150	370	260
(29) Calf m.	70	130	180	260

a estimate based in the data from Melia et al.

## 4 Discussion

The study presented in this article included different methodological and organizational factors that influenced the course of the experiments and the quality of the limits. The most relevant factors and considerations preceding them are analyzed in the following to elucidate and evaluate the approaches taken. Finally, the results obtained from the statistical analysis are compared to results from similar studies.

### 4.1 Scope and Objectives

The objective of this study was to provide experimentally determined biomechanical limits for safe pHRI in order to protect humans from serious consequences of impacts or pinching contacts with cobots. We are confident that limits reflecting the pain threshold fulfill the objective well, since the onset of pain is the first form of biomechanical stress when human tissue is subjected to external forces. Our aspiration was, however, bounded by a variety of constraints. For instance, the validity of the limits is tied to the body locations we have tested in the study. Their choice traces back to the body locations listed in ISO/TS 15066, which actually only covers a small part of the human body. How reliably the limits reflect the mechanical tolerance of entire body regions is uncertain. Research on pain sensitivity maps and the innervation of human skin indicates that pain thresholds vary substantially, even in small body regions (van Hees and Gybels, 1981; Schmidt et al., 1997; Fernandez-de-las Penas et al., 2009; Binderup et al., 2010). Given these findings, it must be assumed that the limits are only valid for their associated body locations, but not for entire body regions.

In addition to the overall validity of the limits, it must be mentioned again that medical concerns precluded any testing of the neck muscle. To fill this gap, we decided to estimate the missing limits based on similar distributed pain thresholds of another body location. The data of Melia et al. (2019) indicate that neck muscle and temple have similar distributions. The mean value of the neck muscle is, however, 1.2 times the mean value of the temple (21.7 ± 10.4 N/cm^2^ vs. 17.9 ± 10.0 N/cm^2^). Assuming the trend also applies to blunt contacts and impacts, we can multiply the limits for the temple by 1.2 to achieve sufficiently accurate limits for the neck muscle. The estimates for the neck muscle are already included in Table 8.

Regardless the limits’ validity and completeness, the question remains if the onset of pain is a reasonable stress level to protect humans from hazardous contacts with cobots. From a biomechanical view point, the onset of pain is the lowest measurable threshold beyond discomfort. Their limits are significantly lower than every other quantifiable stress level (Behrens and Elkmann, 2021). We, therefore, expect that pain-onset limits will ultimately lead to slow cobots. Here, the question arises whether pain-onset levels are too conservative and justify the expected loss of robot productivity. Initial findings from other human-subject studies suggest that the limits for the onset of blunt injuries (defined by the appearance of bruising or swelling) are approximately three times as high as the limits for the onset of pain (Desmoulin and Anderson, 2011; Behrens and Elkmann, 2014; Behrens and Elkmann, 2021). The disparity between pain- and injury-onset limits is considerable high and should start a community-wide discussion about incorporating further and slightly severe levels of biomechanical stress (e.g., the onset of injury) into future standards.

### 4.2 Measurement and Procedural Errors

Manual readings were unnecessary during the experiments, since a fully automated measurement software controlled the data recording, conversion and storage. All instruments were properly calibrated prior to the tests according to the manufacturers’ specifications. Section 2.2 had already given the maximum relative error values for the sensors that include all sources of error across the entire measuring chain from signal acquisition to conversion.

Procedural errors arise mostly from slight misalignments of the pendulum due to differences in the anatomical shape of the subjects’ body locations. These misalignments made it difficult for the experimenter applying the impact loads exactly perpendicular to the body surface. Body locations (5) C7, (6) shoulder joint, (8) sternum, (9) pectoral m., (11) pelvic bone, and (27) kneecap have shown the highest variability. The localization of the body locations constituted another potential source of error, especially when working with overweight subjects. Their high fat content under the skin complicated finding anatomical landmarks by palpation. Vibrations from the impact were another source of error, since they could cause the body parts to slip out of place. To avoid such misalignments, the experimenter used various means to rigidly secure the body parts.

Another critical factor might be the subjects’ ability to sense their individual pain threshold during the load tests. Although the subjects were familiarized with sensing the onset of pain and distinguishing it from discomfort, it is not clear how precisely they assessed themselves and whether they stopped the tests too early or too late. Moreover, some subjects might have tensed their muscles right before the load was applied, which can have a significant effect on the results (Dhaliwal et al., 2002). As a measure to maintain the moment of surprise, subjects wore sleep masks and headphones.

It should also be noted that the subjects signaled the occurrence of pain in different ways. In the tests with the algometer, the subjects pressed a hand switch the moment they began to sense pain. The signal from the hand switch was then used to determine the exact contact force or pressure that ended up causing pain. We can presume that this technique, which was also applied in the studies of Yamada et al. (1996), Saito and Ikeda (2005), or Melia et al. (2019), is sufficiently accurate for the study’s purpose, since the moment when the subject presses the switch is likely to be very close to the moment when the subject feels an initial pain. In the impact tests, the subjects had to say “stop” when the last impact caused pain. In fact, an impact’s effect on the human body is transient and cannot be intensified during the contact. The only option we had to provoke pain was to increase the intensity of the load gradually over multiple impact tests and to stop once the last impact causes the subject pain. The true threshold lies, therefore, between the maximum forces or peak pressures measured from the last two tests, why the results are interval-censored. We estimated the true but unknown critical loads using the technique of midpoint imputation (Sun, 2006) in order to process them in the same way as we did for the observations from the algometer tests. Indeed, it could happen that the midpoint imputation lead to under- or overestimated observations. To compensate the effect of wrong estimates on the limits, we have increased the impact velocity in small steps of 0.05 m/s (0.01 m/s for tests on the head). Because of Eq. 4, it was expected that the maximum force would also increase in such small increments.

As mentioned in Section 2.2.3, the pressure film used in the load tests has blind spots which a spline interpolation filled with estimates. This technique was first utilized by Melia et al. (2019), who used the same contact body and pressure film. However, Melia et al. do not mention if they have validated the technique and how reliable its estimates are. We, therefore, conducted a brief finite-element analysis (FEA) to analyse how the force is distributed within the rounded corners of F-Q10, which were not covered by the pressure film. It turned out that meaningful results from a FEA require complex models and considerable computational effort. Unfortunately, the efforts were not covered by the available budget, why we could not proceed with the FEA we have started. It is, therefore, uncertain if the estimates from the interpolation technique are correct. As a measure to mitigate the effect of wrong estimates, a scaling operation was applied after the interpolation. The operation ensured that the pressure values sum up to the contact force.

### 4.3 Experimental Approach

The study employed various methodologies and materials to emulate impact and pinching loads applied through blunt and semi-sharp surfaces. Their conformance with the ability of robots to cause biomechanical stress to humans cannot taken as granted. Particular attention must be paid to the testing systems. Both, the algometer and pendulum, were designed similarly to systems used in other studies (Patrick, 1981; Yamada et al., 1996; Dhaliwal et al., 2002; Saito and Ikeda, 2005; Muggenthaler et al., 2006; Melia et al., 2014, 2019). However, this does not apply to the contact bodies. Only contact body F-Q10 (see Figure 4) was fully based on an existing design (Melia et al., 2014; Melia et al., 2019). Unlike F-Q10, F-C30 was created by us without having any guidance on suitable parameters for blunt contact bodies. Hence, the quality of F-C30 cannot be evaluated and requires further investigations. It is also uncertain how relevant F-Q10 and F-C30 are for pHRI in industrial environments. Our study had, however, not the aspiration to pick contact bodies which are relevant for pHRI, but contact bodies that enable us to factor in the spatial components of neural pain mediation of humans as elaborated by Behrens and Elkmann (2021).

Each body part was tested several times in a row with both testing systems. To avoid the repeats affecting the subjects’ thresholds, we included an idle time of 45 s between consecutive tests with the alogmeter, which is more time the body locations needs to fully recover (Chesterton et al., 2003). The idle time between two subsequent impact tests on the same body location was approximately 5 s. Given the findings from other studies, there is no clear evidence that multiple transient loads applied in such short times affect an individual’s pain threshold (Brennum et al., 1989; Kosek et al., 1993; Isselée et al., 1997; Chesterton et al., 2003).

The algometer was only equipped with a single-axis force sensor. It is, therefore, impossible to establish a connection between shear forces and the onset of pain or to examine if shearing is actually responsible to cause pain. Given the findings from other pain studies and the long-established techniques of algometry (Haslam, 1967; Fischer, 1986, 1987; Jensen et al., 1986, 1992; Buchanan and Midgley, 1987; Brennum et al., 1989; Takala, 1990; Hogeweg et al., 1992; Kosek et al., 1993; Lee et al., 1994; Fillingim and Maixner, 1995; Yamada et al., 1996; Isselée et al., 1997; Isselee et al., 1998; Fredriksson et al., 2000; Chesterton et al., 2003; Fernandez-de-las Penas et al., 2009; Finocchietti et al., 2011; Finocchietti et al., 2012), it is most likely that normal forces are the primary reason of mechanically evoked pain.

The preliminary limits in ISO/TS 15066 for impacts apply to unconstrained spatial conditions, but we had to secure the body parts during the tests to avoid misalignments. Nevertheless, the impacts applied in the experiments can be compared to those in which a robot without a fast collision detection or sophisticated safety controller hits a free moving part of the human body. The fact that the pendulum recoiled after hitting the subject emulates an impact under unconstrained spatial conditions, albeit from a different perspective. An accurate reproduction of free impacts would have required matching the colliding masses exactly to the apparent mass of the tested body part. Only then would the situation exactly correspond to the conservative case in which an imaginary robot of infinite mass collides with a freely moving human body part (Haddadin et al., 2009). The efforts required to adjust the pendulum mass precisely to the mass of the body part under test are, however, essentially high.

None of the tests has caused any injuries to any of the subjects, even slight ones (e.g., skin damage). Moreover, none of the subjects has observed an increase in tenderness to mechanical pressure. We can, therefore, conclude, that the loads we have applied were in the range below the injury onset.

### 4.4 Limitations of the Statistical Analysis

To reduce the influence of inter-rater errors, all tests were executed by one single experimenter. The decision to have only one person responsible must be seen as critical because it impeded to trace this type of error. As a countermeasure, our study protocol described the steps of all procedures in detail and does not permit deviations.

Inter-subject variability could not be analyzed in this study, because this would have required more repeated measurements per subject, executed in separate sessions. Unfortunately, with the available budget, it was not possible to hold more sessions without reducing the number of subjects. In favor of larger samples, we preferred to maximize the number of subjects. As recent findings of Liew et al. (2021) indicate, our decision not to increase the number of repeats seems to barely affect the results we have achieved. According to their findings, the variance in a subject’s pressure pain threshold measured in separate sessions can be expected to be irrelevant compared to the inter-subject variance. The intra-class coefficients presented by Park et al. (2019) draw a similar picture. In this light, we can presume that the inter-subject variability is of minor relevance and would have contributed little to the study’s objectives.

The effect of gender on pain thresholds could not be confirmed for all body locations and conditions. This outcome is in accord with other studies (Lee et al., 1994). According to Riley et al. (1998), the groups tested must include at least 41 males and 41 females to identify a gender difference with a power of 0.70. At this point, it is important to highlight that the objective of our study was not to examine the gender difference, but to determine biomechanical limits for safe pHRI. Nevertheless, in order to take the effect into account, we extended a statistical model Eq. 13 that allows for adjusting the limits to a group with a specific percentage of males. In our opinion, the covariate in the extended model is only relevant if the effect of the gender on the pain thresholds and thus on the limits is significant for at least two different testing conditions (see Table 5). Otherwise, the effect must be neglected by setting *β*_1_ = 0.

### 4.5 Data Comparison

In our study, we also tested a control group (G5) of eleven subjects with pinching loads applied through F-Q10. The results from the control group can be directly compared to the results presented by Melia et al. (2019) and Park et al. (2019), since both studies utilized an identical setup.

In their recent article, Melia et al. (2019) present the peak pressures obtained for the 80th percentile (P80) of their empirically distributed observations. It seems that their P80 values are actually P90 values since the P80 values in Melia et al. (2019) are identical with the P90 values, which the same authors have presented in a publicly available report (Muttray et al., 2014). To compare the results from our study to those of Melia et al., we calculated P90 values with Eq. 13 for a group consisting of 57% males and 43% females (*x*_*G*_ = 0.57), which is precisely the gender distribution of the group examined by Melia et al. Table 9 presents the P90 values from both studies (2nd and 3rd column). For a better comparison, we define the relative deviation *ϵ*_*i*_

$$\epsilon_{i}=1-\frac{{\hat{y}}_{0.9}^{Si}}{{\hat{y}}_{0.9}}\;,$$ (15)

where ${\hat{y}}_{q}$ denotes a quantile value from our study and ${\hat{y}}_{q}^{Si}$ a quantile value from the other study, both calculated for *q*. Index *i* ∈ {1, 2} indicates the study. The deviation values *ϵ*_1_ obtained from our P90 values and the ones from Melia et al. (*i* = 1) are also listed in Table 9 (4th column) and indicate that the difference between the values is remarkably high. The average deviation is −181% in an asymmetric range of −584 to 8%. The deviation is especially obvious for body locations with a distinct layer of soft tissue which usually tend not to create regions of high pressures. Melia et al. have identified, for instance, a threshold of 335 N/cm^2^ for (10) abdominal muscle, which must approximately correspond to a maximum contact force of 335 N if we presume that at least 1 cm^2^ of F-Q10’s face transmits the force and that the highly compliant tissue on the abdomen tends to distribute the force evenly. Melia et al. report, however, a force-based threshold of 36 N, which is an order of magnitude lower than the expected 335 N.

**TABLE 9:** Comparison of the P90 values from Melia et al. (2019) (S1, 57% males) and Park et al. (2019) (S2, 100% males) with the values from control group G5; *ϵ*_*i*_ is the relative variance between the limits; *k* is the coefficient of the Pearson correlation for the data from control group G5 (pressure mapped over force).

Body part	P90, *x*_*G*_ = 0.57			P90, *x*_*G*_ = 1			
	S1^a^	G5^a^	*ɛ*_1_	S2^a^	G5^a^	*ɛ*_2_	*k*
(1) Forehead	176	164	−0.07	150	230	0.35	0.95
(2) Temple	172	81	−1.12	—	91	—	0.95
(3) Masticatory m.	182	51	−2.57	—	60	—	0.83
(5) C7	303	90	−2.37	197	108	−0.82	0.50
(6) Shoulder joint	224	92	−1.43	120	108	−0.11	0.94
(7) L5	268	104	−1.58	134	134	0.00	0.98
(8) Sternum	165	79	−1.09	—	104	—	0.96
(9) Pectoral m.	266	70	−2.80	110	70	−0.57	0.93
(10) Abdominal m.	335	49	−5.84	122	71	−0.72	0.94
(11) Pelvic bone	255	277	0.08	—	454	—	0.93
(12) Deltoid m.	277	109	−1.54	—	142	—	0.97
(13) Humerus	295	64	−3.61	—	82	—	0.88
(14) Radial bone	238	111	−1.14	—	144	—	0.96
(15) Forearm m.	251	83	−2.02	—	122	—	0.91
(16) Arm nerve	289	93	−2.11	92	107	0.14	0.97
(17/18) Forefinger pad	402	117	−2.44	236	153	−0.54	0.77
(19/20) Forefinger DIP	332	213	−0.56	—	261	—	0.89
(21) Thenar eminence	261	86	−2.03	172	98	−0.76	0.93
(22/23) Palm	356	81	−3.40	146	110	−0.33	0.96
(24/25) Back of the hand	279	238	−0.17	288	260	−0.11	0.62
(26) Thigh muscle	404	110	−2.67	121	144	0.16	0.96
(27) Kneecap	354	175	−1.02	—	264	—	0.97
(28) Middle of shin	294	222	−0.32	188	367	0.49	0.70
(29) Calf m.	299	116	−1.58	127	148	0.14	0.97

a pressure-based pain thresholds in [N/cm^2^] for the 90th percentile and gender distribution *x*_*G*_

The P90 values reported by Park et al. (2019) (5th column in Table 9) were obtained from a group that only consists of male subjects. They must be, therefore, compared to limits for *x*_*G*_ = 1 (6th column). Applying Eq. 15 for *i* = 2 reveals significantly smaller and more symmetric deviations ranging from −82 to 49% (average is −20% and thus close to zero). We can conclude that the pain thresholds from Park et al. are much closer to the pain thresholds we have obtained. The authors do not mention if they also had to fill any blind spots in the pressure images with estimates (see Section 2.3). Their peak pressures must, therefore, be considered as results obtained without interpolation. Since an interpolation tends to reduce the peak pressures when the pressure values are calibrated to the contact force, it can be expected that the relative differences are actually smaller.

The last column in Table 9 shows the Pearson coefficient *k*, which is a quality measure for the correlation of maximum contact force and peak pressure. It can be used to evaluate the quality of the peak pressure obtained from control group G5. As expected, all values of *k* indicate a strong correlation and reflect the causal relationship between both quantities.

## 5 Conclusion

The objective of our study was to determine biomechanical limits for safe human-robot interactions in which potentially hazardous impacts and pinching contacts can occur due to human error or technical failures. To keep with ISO/TS 15066, our work focuses on pain-onset limits for 29 body locations and four contact events, varying in load type and contact type. We decided to collect the data for the limits in experiments with human-subjects and not to derive them from literature data. Altogether 112 subjects, organized in four regular test groups and one control group, participated in the study. A conventional and expanded model based on the log-logistic CDF were developed that enable us to calculate the desired limits from the experimentally gathered data. The models and their parameters can now be used to determine pain-onset limits for an arbitrary quantile of a group with a given distribution of males and females of employable age.

In addition to the limits in Table 8, the data of this study can be used to calculate biomechanical response corridors for the body locations tested. Such corridors are especially helpful either to design proper instruments for evaluating the intensity of human-robot impacts through measurement (Falco et al., 2012; Huelke and Ottersbach, 2012), or to create accurate models for analyzing them in simulations. Our future work will specifically focus on such models, since they have great potential to replace the measurement-based approaches. Moreover, the experimental data make it possible to analyze other output variables, such as maximum energy or power density. Energy-based limits are of particular interest in robotics (Haddadin et al., 2012; Lee et al., 2013), since they can be easily converted into velocity limits used to program safely working cobots.

As discussed in Section 4.1, the use of pain-onset limits is possibly not ideal, but will most likely prevent cobots from causing injuries. At least, our tests did not cause even slight injuries (e.g., visible skin damage) to any of the subjects. Nor did any subject observe a change in tenderness during palpation. From a scientific standpoint, the use of pain-onset limits raises the question about the limits’ objectiveness, because it is well examined that emotional and neural factors can affect an individual’s pain thresholds. To date, only one pilot study has examined the onset of injury with multiple subjects (Behrens and Elkmann, 2014).

## Data Availability

The datasets presented in this article are not readily available because the full dataset belongs legally to the study’s contractor. Requests to access the datasets should be directed to roland.behrens@iff.fhg.de.
